# Generation and characterization of a recombinant Newcastle disease virus expressing the red fluorescent protein for use in co-infection studies

**DOI:** 10.1186/1743-422X-9-227

**Published:** 2012-10-03

**Authors:** Jinnan Li, Haixia Hu, Qingzhong Yu, Diego G Diel, De-shan Li, Patti J Miller

**Affiliations:** 1 USDA-ARS, Southeast Poultry Research Laboratory, 934 College Station Road, Athens, GA 30605, USA; 2 Heilongjiang Fisheries Research Institute, Harbin, 150070, China; 3 College of Animal Science and Technology, Southwest University, 2 Tiansheng Road, Chongqing, BeiBei District, 400715, China; 4 College of Life Sciences, Northeast Agriculture University, Harbin, 150030, China

**Keywords:** NDV, Co-infection, Super infection, Interference

## Abstract

**Background:**

Many viruses have evolved multiple strategies to prevent super infection of host cells by more than one virion. This phenomenon, known as super infection exclusion, may play an important role on virus evolution because it can affect the frequency of reassortment and/or recombination. Newcastle disease virus (NDV), a negative sense single-stranded RNA virus, is characterized by its continuous evolutionary dynamics and by a low frequency of recombination events. However, the mechanisms that contribute to the low recombination rates on NDV are still not completely understood.

**Methods:**

In this study we assessed the ability of two NDV strains (LaSota and B1) to super infect host cells *in vitro*. We generated a recombinant NDV strain LaSota expressing the red fluorescent protein (RFP) and used it in co-infection assays with a related NDV strain B1 expressing the green fluorescent protein (GFP). DF-1 cells were inoculated with both viruses at the same time or at different intervals between primary infection and super infection.

**Results:**

When both viruses were inoculated at the same time point, a 27% co-infection rate was observed, whereas when they were inoculated at different time points the super infection rates decreased to levels as low as 1.4%.

**Conclusions:**

These results indicate that although different NDV strains can co-infect host cells *in vitro*, the super infection rates are low, specially as the time between the primary infection and super infection increases. These results confirm the occurrence of super infection exclusion between different strains of NDV.

## Introduction

Newcastle disease (ND) is one of the most important diseases of poultry, causing significant economic losses to poultry producers around the world [1]. The etiologic agents of ND is Newcastle disease virus (NDV), or avian paramyxovirus type 1 (APMV-1), an enveloped, non-segmented, single-stranded, negative sense RNA ([−]ssRNA) virus of the genus *Avulavirus* family *Paramyxoviridae*[1]. The NDV genome is approximately 15.2 Kb in length and contains six genes encoding for the nucleoprotein (NP), the phosphoprotein (P), the matrix protein (M), the fusion protein (F), the hemagglutinin-neuraminidase (HN), and the RNA-dependent RNA polymerase (L). At least one additional protein (V) is produced through editing of the phosphoprotein messenger RNA (mRNA) [1,2].

NDV is known for its continuous evolutionary dynamics, with different isolates undergoing simultaneous evolutionary changes around the world [2,3]. This evolutionary dynamic is likely resultant from the lack of proofreading activity of the viral RNA dependent RNA polymerase [4,5]. While mutations introduced during virus replication seem to play a major role on NDV evolution, recombination has only been rarely reported [3,6-9]. In general, the low frequency of recombination in [−]ssRNA viruses reflect specific aspects of their life cycle, including their genome organization and the mechanisms involved in transcription and replication [10]. Other aspects the infection cycle and virus-host interactions may also affect the recombination rates in RNA viruses [10]. For NDV, however, the strategies/mechanisms that contribute to the low frequency of recombination are not completely understood.

Many enveloped viruses have evolved mechanisms to prevent infection of host cells by more than one virion [11-13]. This phenomenon, known as super infection exclusion, likely affects the frequency of recombination within a viral population because co-infection of a cell by related viruses is required for the exchange of genetic material. In this study we assessed the ability of two NDV strains (LaSota and B1) to super infect host cells *in vitro*. We generated a recombinant NDV strain LaSota, encoding the red fluorescent protein (RFP; rLS-RFP) and used it in co-infection assays with a related strain of NDV (B1) expressing the green fluorescent protein (GFP; rB1-GFP).

## Methods

### Cells and viruses

HEp-2 (ATCC, CCL-23), Vero (ATCC, CCL-81) and DF-1 (ATCC, CRL-12203) cell lines were cultured in Dulbecco’s Modified Eagle Medium (DMEM, Invitrogen, Carlsbad, CA) supplemented with 10% fetal bovine serum (FBS, Invitrogen) and antibiotics and antimycotic (100U/ml Penicillin, 100 μg/ml Streptomycin, 0.25 μg/ml Amphotericin B, Thermo Scientific), and maintained at 37°C with 5% CO_2_. DF-1 cells were supplemented with 10% allantoic fluid (AF) (from specific pathogen free [SPF] embryonated chicken eggs) during all virus infections. The NDV strain LaSota was obtained from ATCC and the recombinant NDV strain B1 expressing the green fluorescence protein (rB1-GFP) was kindly provided by Dr. Peter Palese, Department of Microbiology, Mount Sinai University [14]. All viruses were propagated in 9-day-old SPF embryonated chicken eggs and the virus titres were determined by limiting dilution, calculated according to the Spearman and Karber’s method, and expressed as Log_10_ tissue culture infectious dose 50 (TCID_50_)/ml. The modified vaccinia virus Ankara/T7 (MVA/T7) was used in the infection/transfection experiments to rescue the recombinant virus NDV-LS/RFP.

### Generation of the recombinant NDV-LaSota containing the RFP gene

**Figure 1:**
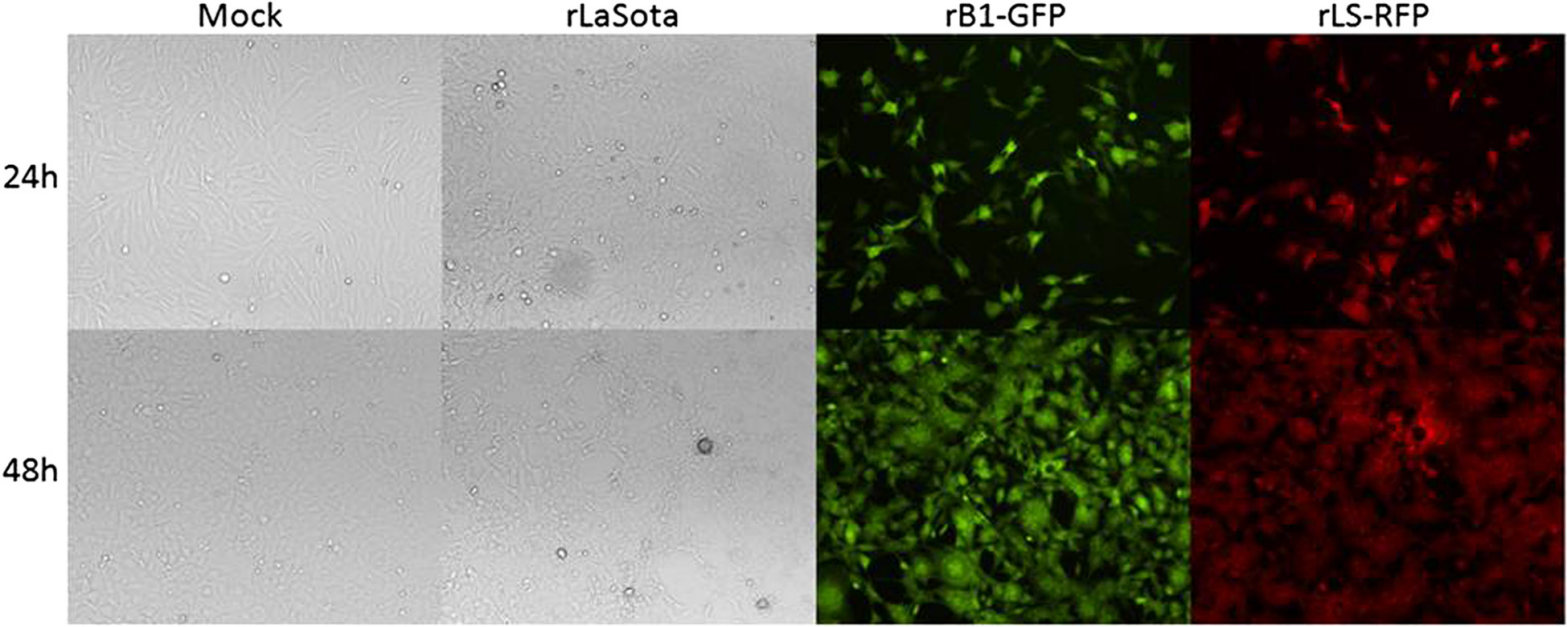
**Cytopathic effect and expression or RFP and GFP in rLS-RFP- and rB1-GFP-infected DF-1 cells.** Cells were inoculated with each virus at a MOI of 0.01 and examined under a fluorescence microscope 24 and 48 h pi.

The recombinant NDV LaSota expressing the red fluorescent protein (RFP) (rLS-RFP) was generated from the backbone of the vector pLS/aMVP-C G [15]. The open reading frame of the RFP was inserted between the fusion (F) and hemagglutinin-neuraminidase (HN) genes as an extra transcriptional unit (Figure 1). Initially, an intermediate plasmid containing the aMPV-C G gene and its flanking regions (including the NDV gene start [GS] and gene end [GE] sequences) was generated by PCR amplification (primers: LSFF-5’-TCAGATGAGAGCCACTACAAAAATGTGAGC-3’ and LS6682R-5’ACTCAAGGGCCACTTG CT-3’) and cloning of the PCR product into the TOPO TA® vector (Invitrogen). The aMPV-C G protein coding sequence was replaced with the open reading frame (ORF) of the RFP gene using the In-Fusion® PCR Cloning kit (Clontech) and two sets of primers (including LS-NI-up-5’-CTTGCACCTGGAGGGCGCCAAC-3’ and LS-NI-down-5’-CGTACACAGATGAGGAACGAAGGTTTCCCT-3’ to linearize the vector, and NI-RFP-F-5’CCCTCCAGGTGCAAGATGGCCTCCTCCGAGGAC-3’ and NI-RFP-R-5’-CCTCATCTGTGTACGCTACAGGAACAGGTGGTG-3’ to amplify the RFP ORF from the pCMV-DsRed-Express plasmid, Clontech). The coding sequences of the RFP and its flanking regions were PCR amplified (primers: LSFF and LS6682R, described above) and cloned into the vector containing the full-length LaSota genome using the InFusion PCR cloning kit and specific primers (LS-vec-F-up-5’-GTGGCTCTCATCTGATCTAGAGTATTATTC-3’ and LS-vec-6682-down-5’-5’AAGTGGCCCTTGAGTCTCCGTTGGCATTGTTA-3’). The resultant plasmid, designated pLS-RFP, was amplified in Stbl2 competent cells (Invitrogen) and purified using a QIAprep Spin Miniprep kit (Qiagen).

Rescue of the rLS-RFP was performed in HEp-2 cells according to standard protocols [15]. HEp-2 cells were seeded on a six-well plate (1×10^6^ cells/well), infected with MVA/T7 at a multiplicity of infection (MOI) of 3, and one hour later transfected with the plasmid pLS-RFP (2 μg), and the helper plasmids pTM-NP (1 μg), pTM-P (0.5 μg), and pTM-L (0.1 μg) using Lipofectamine 2000™ (Invitrogen). Seventy-two hours post-infection/transfection cells were harvested and subjected to three freeze and thaw cycles. Approximately 300 μl of the cell lysate were inoculated into the allantoic cavity of 9-day-old SPF embryonated chicken eggs for amplification of the recombinant virus. Rescue of rLS-RFP was confirmed by hemagglutination (HA) and fluorescence assays.

### Biological characterization of rLS-RFP

The pathogenicity of the rLS-RFP virus was assessed by standard mean death time (MDT) and intracerebral pathogenicity index (ICPI) tests [16]. To compare the cytopathic effect (CPE) induced by rLS-RFP, the parental NDV-LaSota and the rB1-GFP, DF-1 cells were infected with each virus (MOI=0.01) and evaluated under a light and a fluorescence microscope at 24 and 48 hours post infection (pi). Growth characteristics of rLS-RFP, NDV-LaSota, and rB1-GFP were assessed in DF-1 cells. Cells were infected with rLS-RFP, NDV-LaSota, or rB1-GFP (MOI=0.01), harvested at 0, 12, 24, 36 and 48h pi and subjected to three freeze and thaw cycles. Viral titres were determined by limiting dilution, calculated according to the Spearman and Karber’s method, and expressed as Log_10_ TCID_50_/ml.

### Super infection assays

The ability of rLS-RFP and rB1-GFP to super infect host cells was assessed in a chicken fibroblast cell line (DF-1). Initially, rLS-RFP and rB1-GFP were mixed (MOI=3 of each virus), and inoculated in DF-1 cells. After 1h of adsorption at 37°C the virus inoculum was removed, cells were washed 1X with DMEM, and complete culture media was added to the cells. At 12h pi cells were stained with HCS Nuclear Mask Blue (Invitrogen) and examined under a fluorescence microscope (Nikon, Eclipse T*i*, Melville, NY). Pictures were taken at 20X magnification and the numbers of red-, green-, or yellow-fluorescent cells (red + green) were determined using the ImageJ (version 1.46) software (NIH, http://rsbweb.nih.gov/ij/index.html). The numbers of cells infected with rLS-RFP, and rB1-GFP, or co-infected with both viruses (yellow-fluorescent cells) were expressed as percentage of infected cells. At least three fields were randomly selected to calculate the mean infectivity and super infection percentages.

To assess the effect of the time of super infection in the ability of rLS-RFP and rB1-GFP to co-infect host cells, DF-1 cells were culture in 12-well plates and inoculated with both viruses at different intervals between the primary infection and the super infection. Cells were inoculated with rLS-RFP and rB1-GFP (MOI=3) and super infected at 1 and 3h pi with rB1-GFP or rLS-RFP (MOI=3), respectively. In a second experiment, cells were inoculated with rLS-RFP or rB1-GFP (MOI=1) and super infected at 12, and 24h pi with rB1-GFP or rLS-RFP (MOI=3), respectively. At 24h post super infection cells were stained with HCS Nuclear Mask Blue (Invitrogen), and examined under a fluorescence microscope (Nikon, Eclipse T*i*, Melville, NY). The numbers of red-, green-, and yellow-fluorescent cells were determined as described above. In addition, Vero cells were inoculated with rLS-RFP or rB1-GFP (MOI=3) and super infected at 3h pi with rB1-GFP or rLS-RFP (MOI=3), respectively. At 24h post super infection cells were examined under a fluorescence microscope (Nikon, Eclipse T*i*, Melville, NY). Statistical analysis was performed with the two-tailed paired Student’s *t*-test, and *p* values < 0.05 were considered statistically significant.

## Results

### Construction and characterization of recombinant NDV LaSota expressing the red fluorescent protein (rLS-RFP)

The recombinant NDV LaSota encoding the red fluorescent protein (RFP) was constructed using reverse genetic approaches. The cDNA clone pLS/aMPV-C G [15], containing the complete genome sequences of NDV strain LaSota and the coding sequences of the G protein of avian metapneumovirus type C, was used as backbone to generate the rLS-RFP virus. The open reading frame of the aMPV G protein (1,758 nt) was replaced by the coding sequence of the RFP (678 nt) using the In-Fusion cloning strategy. The rLS-RFP was rescued in HEp-2 cells and propagated in 9-days-old embryonated chicken eggs. DNA sequencing of regions flanking the inserted gene confirmed the integrity of NDV LaSota and of RFP sequences in the recombinant virus (data not shown).

To determine whether the resultant recombinant virus expresses the RFP, DF-1 cells were infected and examined under a fluorescence microscope. Expression of RFP was observed in cells infected with the rLS-RFP at 24h and 48h post-infection (pi), demonstrating efficient expression of RFP by the recombinant virus (Figure 1). The rB1-GFP virus was used as a positive control for the expression of GFP (Figure 1).

### Biological characterization of the rLS-RFP virus

**Figure 2:**
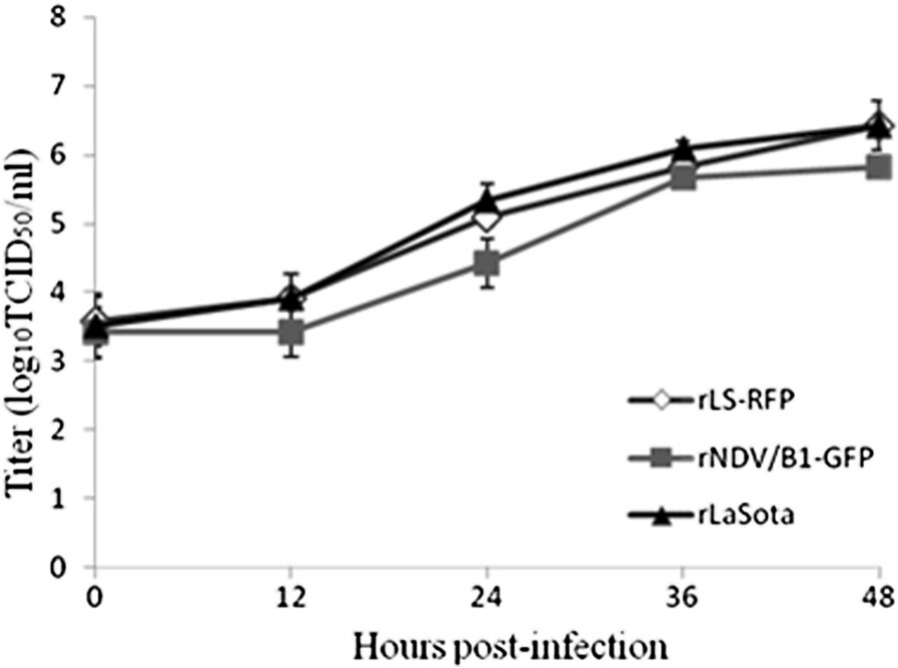
**Replication kineticts of NDV-LaSota, rLS-RFP, and rB1-GFP.** DF-1 cells were infected with rLS-RF, rB1-GFP or LaSota strain (MOI=0.01), harvested at the indicated time points and the virus titres determined by limiting dilution and expressed as Log_10_ TCID_50_/ml. Results shown represent the mean titre calculated from two independent experiments.

The pathogenicity and replication kinetics of rLS-RFP were assessed *in vivo* and *in vitro*, respectively. The intracerebral pathogenicity index (ICPI) and the mean death time (MDT) of rLS-RFP were determined using standard protocols. Insertion of RFP into the NDV-LaSota backbone resulted in a slight attenuation of the recombinant virus, as evidenced by a decreased intracerebral pathogenicity index (ICPI; 0.0) in day-old chicks and a longer mean death time (MDT; 127 h) in embryonated chicken eggs inoculated with rLS-RFP, when compared to those inoculated with the parental virus NDV strain LaSota (Table 1). The rB1-GFP presented a MDT >150 h. No differences in the cytopathic effect (CPE) induced by the parental and recombinant viruses (rLS-RFP and rB1-GFP) were observed in DF-1-infected cells (Figure 1). Additionally, no significant differences in the replication kinetics and viral yields were detected between NDV-LaSota, rLS-RFP, and rB1-GFP in embryonated chicken eggs- or in DF-1-infected cells (Table 1; Figure 2), indicating that insertion of RFP in the F-HN intergenic region did not significantly affect replication of the recombinant virus.

**Table 1:** Biological characterization of the rLS-RFP

**Virus**	**MDT**	**EID**_**50**_	**TCID**_**50**_	**ICPI**
NDV LaSota	110hs	3.20×10^9^	2.37×10^8^	0.15
rLS-RFP	127hs	3.60×10^8^	1.78×10^8^	0
rB1-GFP	>150hs	1.26×10^8^	1.78×10^7^	NT^a^

^a^Not tested.

### Super infection with rLS-RFP and rB1-GFP results in low rates of co-infection *in vitro*

**Figure 3:**
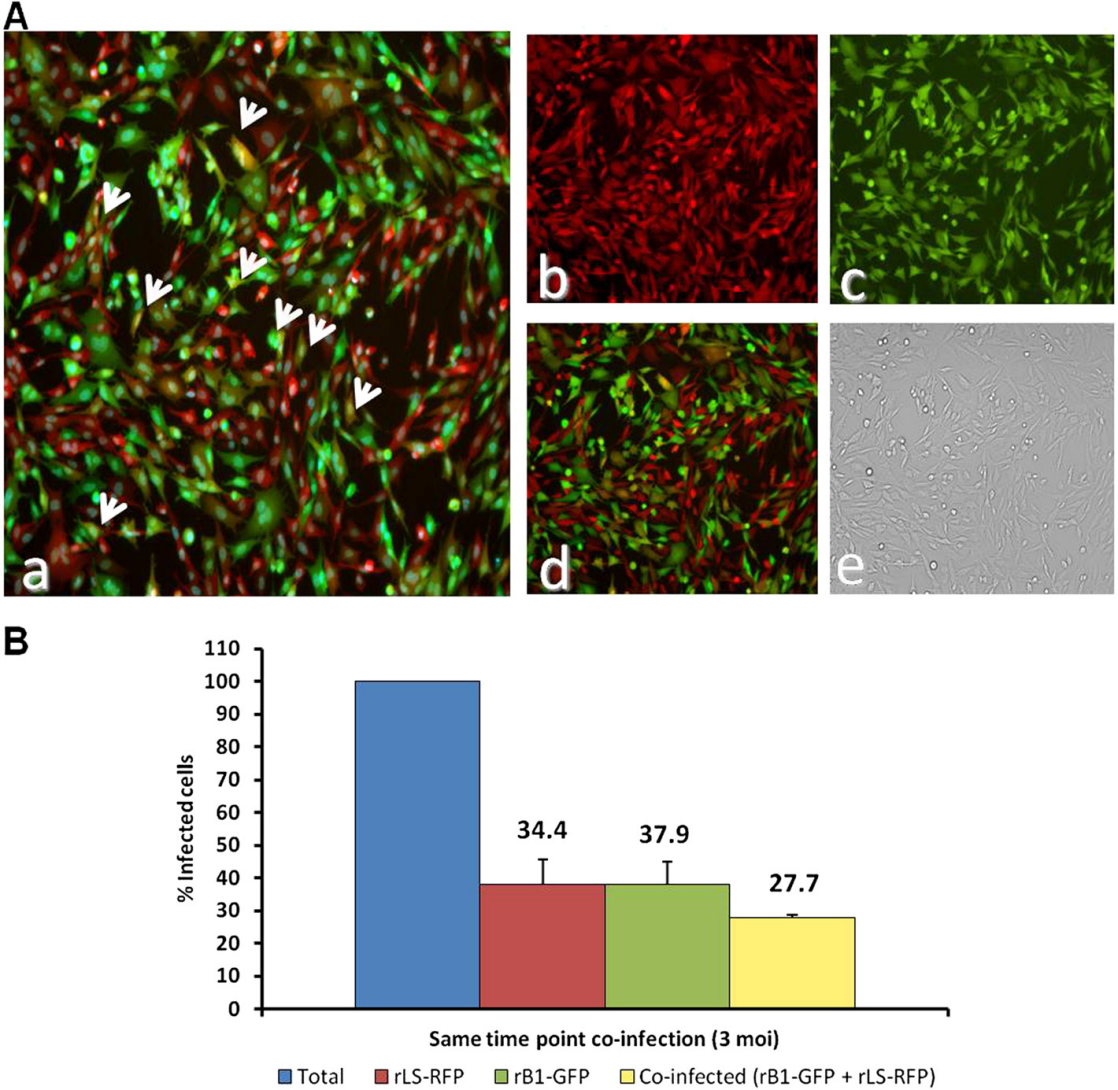
**Co-infection of DF-1 with rLS-RFP and rB1-GFP.** (**A**) Monolayers of DF-1 cells were infected with rLS-RFP and rB1-GFP (MOI=3) at the same time point. At 24h post infection, cells were stained with HCS NuclearMask Blue and examined under a fluorescence microscope. **a**. merged green, red and blue channels; **b**. red channel; **c**. green channel, **d**. merged green and red channels; **e**. bright field. (**B**) Percentage of infected cells. Cells were infected as described in A. Three fields were randomly selected and used to determine the numbers of red-, green- and yellow-fluorescent cells using the ImageJ software. Results shown represent mean percentages of three fields. The percentages of cells infected with each virus (rLS-RFP of rB1-GFP) and the percentage of cells co-infected are expressed as a relative percentage of the total number of infected cells.

The ability of two NDV strains (LaSota and B1) to super infect host cell was assessed *in vitro.* DF-1 cells were co-infected with rLS-RFP (MOI=3) and rB1-GFP (MOI = 3) and the expression of RFP and GFP was examined by fluorescence microscopy. Red- and green-fluorescent cells were observed as early as 12 h pi, confirming that the cells were infected with rLS-RFP or rB1-GFP, respectively (Figure 3A). The co-infection rates were ~27% as evidenced by the presence of yellow-fluorescent cells at 24 h pi (Figure 3A, panel a, and d).

**Figure 4:**
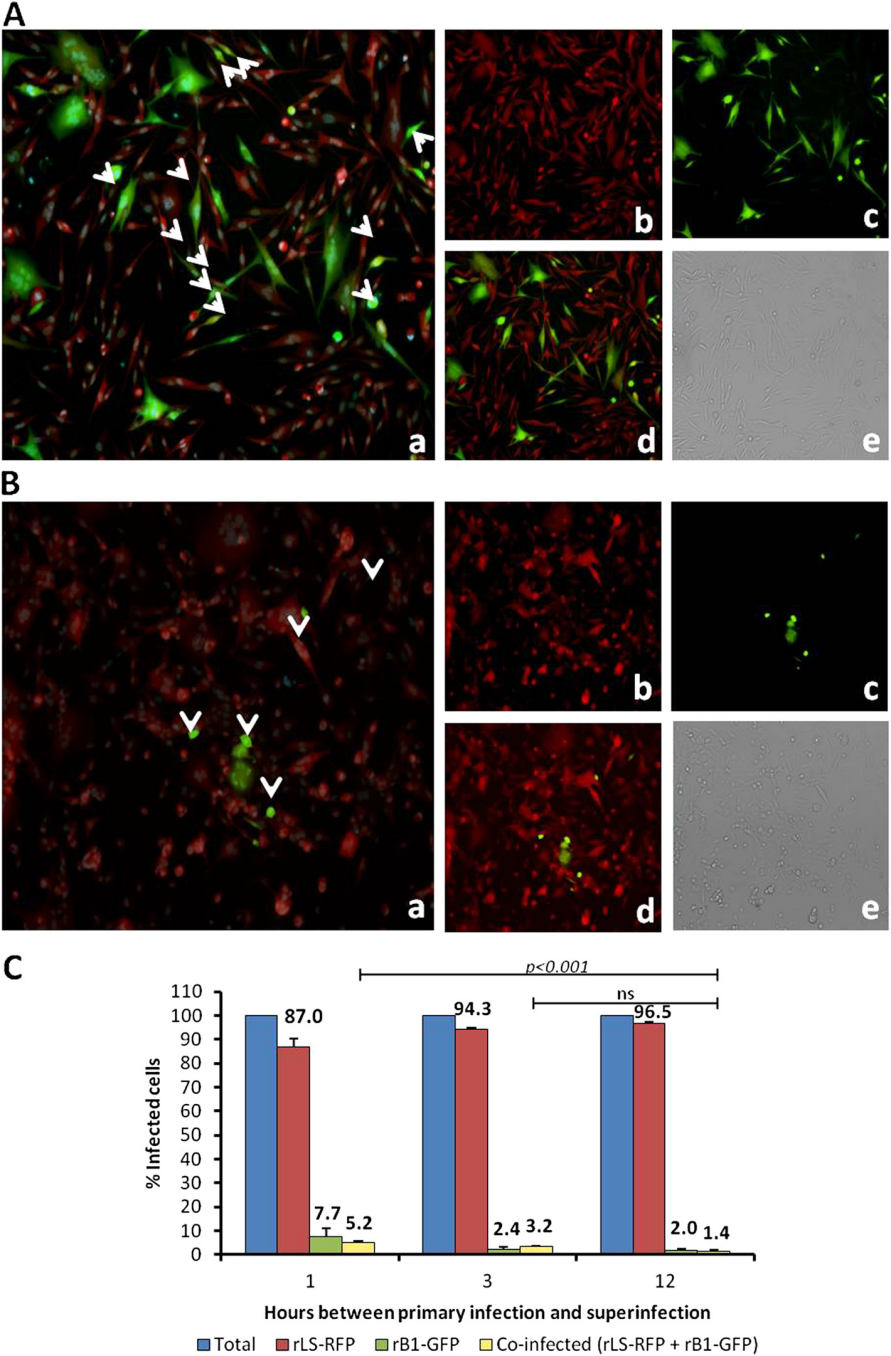
**Co-infection of DF-1 cells with rLS-RFP and rB1-GFP.** (**A**) DF-1 cells infected with rLS-RFP (MOI=3) and super infected with rB1-GFP (MOI=3) at 1 h post primary infection. At 24h post infection, cells were stained with HCS NuclearMask Blue and examined under a fluorescence microscope. **a**. merged green, red and blue channels; **b**. red channel; **c**. green channel, **d**. merged green and red channels; **e**. bright field. (**B**) DF-1 cells infected with rLS-RFP and super infected with rLS-RFP at 12 h post primary infection. **a**. merged green, red and blue channels; **b**. red channel; **c**. green channel, **d**. merged green and red channels; **e**. bright field. (**C**) Percentage of infected cells. Cells were infected as described in A and B. Three fields for each indicated time point were randomly selected and used to determine the numbers of red-, green- and yellow-fluorescent cells using the ImageJ software. Results shown represent mean percentages of three fields. The percentages of cells infected with each virus (rLS-RFP of rB1-GFP) and the percentage of cells co-infected are expressed as a relative percentage of the total number of infected cells.

**Figure 5:**
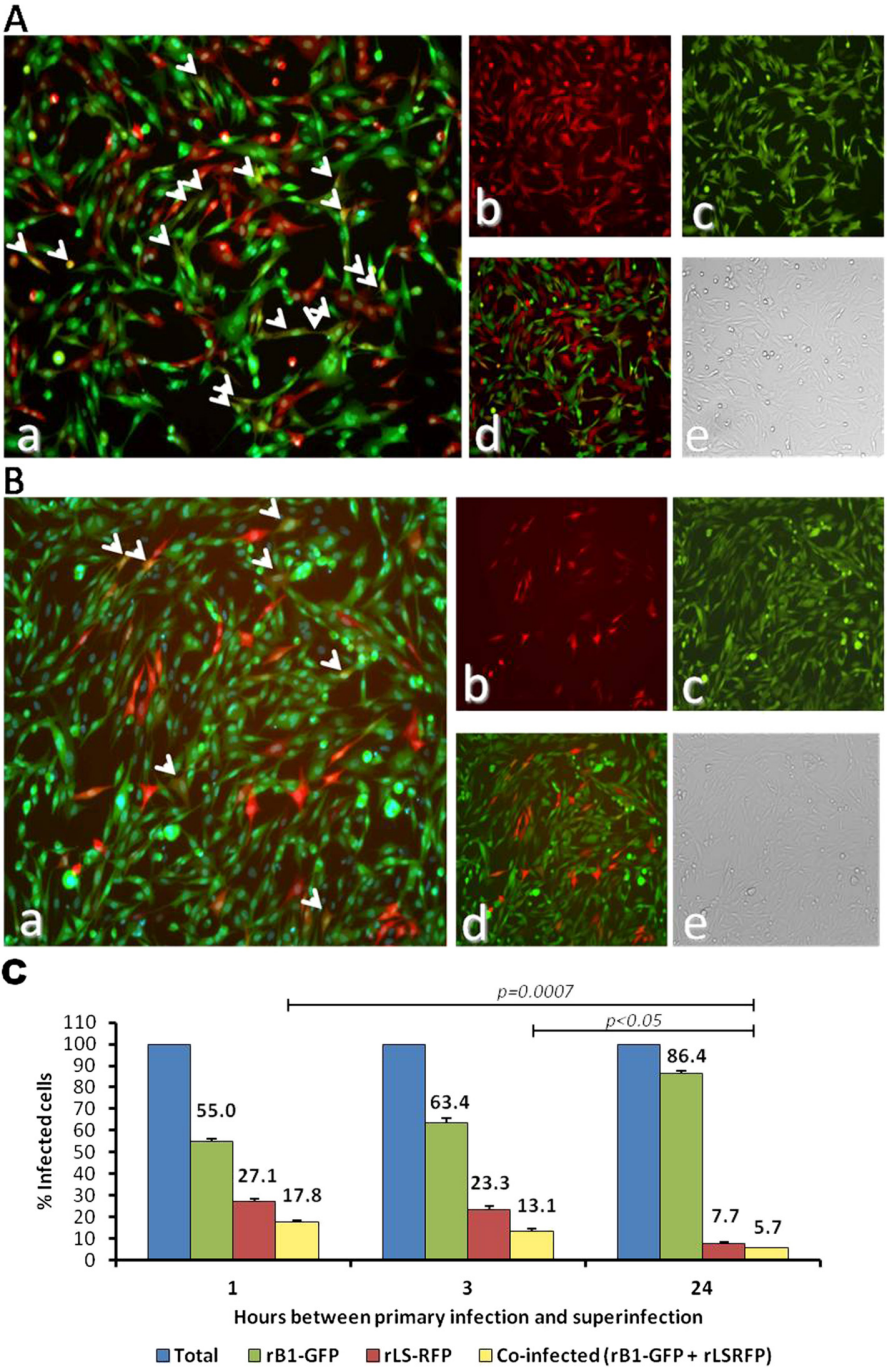
**Co-infection of DF-1 cells with rB1-GFP and rLS-RFP. (A)** DF-1 cells infected with rB1-GFP (MOI= 3) and super infected with rLS-RFP (MOI=3) at 1 h pi. At 24h post infection, cells were stained with HCS NuclearMask Blue and examined under a fluorescence microscope. **a**. merged green, red and blue channels; **b**. red channel; **c**. green channel, **d**. merged green and red channels; **e**. bright field. **(B)** DF-1 cells infected with rB1-GFP and super infected with rLS-RFP at 12 h pi. At 24h post infection, cells were stained with HCS NuclearMask Blue and examined under a fluorescence microscope. **a**. merged green, red and blue channels; **b**. red channel; **c**. green channel, **d**. merged green and red channels; **e**. bright field. **(C)** Percentage of infected cells. Cells were infected as described in A and B. Three fields for each indicated time point were randomly selected and used to determine the numbers of green-, red- and yellow-fluorescent cells using the ImageJ software. Results shown represent mean percentages of three fields. The percentages of cells infected with each virus (rB1-GFP or rLS-RFP) and the percentage of cells co-infected are expressed as a relative percentage of the total number of infected cells.

The effect of the time of infection on the ability of NDV LaSota and B1 to super infect host cells was also investigated. DF-1 cells were infected with rLS-RFP or rB1-GFP and super infected with rB1-GFP or rLS-RFP at different intervals post-primary infection, respectively (1, 3, 12 and 24 h pi). As in the co-infection assay described above, super infection at intervals of 1, 3, 12 and 24 h resulted in low rates of co-infection, confirming the occurrence of viral interference or super infection exclusion among these viral strains [17] (Figure 4A, B, and C; Figure 5A,B and C). Notably, the co-infection rates significantly decreased as the time between primary infection and super infection increased (Figure 4A,B and C; Figure 5A,B and C). To rule out the possibility that the super infection exclusion observed in DF-1 cells was mediated by a host antiviral response induced by the primary infection, we performed the super infection assays in Vero cells which are defective in interferon production [18]. Similar to the results observed in DF-1 cells, infection with one NDV strain (rLS-RFP or rB1-GFP) interfered with the super infection by the other, as evidenced by low rates of yellow-fluorescent cells at 24 h pi (Additional file 1: Figure S1). These results demonstrate the occurrence of super infection exclusion between two different strains of NDV in DF-1 and Vero cells.

## Discussion

In the present study we assessed the ability of two NDV strains (LaSota and B1) to co-infect host cells *in vitro*. Results here revealed that NDV strains LaSota and B1 are able to co-infect one host cell simultaneously. However, the super infection rates are low and they decrease as the time between the primary infection and super infection increases. These findings suggest the occurrence of super infection exclusion during NDV infection.

Super infection exclusion is a phenomenon by which an established virus infection blocks co-infection by a homologous super infecting virus [11]. Here we have shown that primary infection of DF-1 cells with either rLS-RFP or rB1-GFP decreases the co-infection rates by the heterologous strain. Interestingly, the co-infection rates decreased as the time between the primary infection and the super infection increased, corroborating with findings from early studies [17]. Super infection exclusion can occur at three different steps of the virus life cycle, including attachment, entry and/or intracellular replication [11-13]. It has been shown that for the paramyxovirus human parainfluenza type 3 the neuraminidase activity of the HN protein is responsible for blocking super infection by a heterologous strain [13]. Whether NDV HN exerts a similar function in blocking super infection or whether the virus has evolved additional mechanisms to prevent super infection remains to be determined.

Although the biological significance of super infection exclusion for NDV and other paramyxoviruses is unknown, this phenomenon can contribute to the low frequency of recombination observed for these viruses. The limited number of cells that are co-infected during NDV infection may negatively affect recombination rates. As shown here, super infection rates were low even when a high multiplicity of infection (MOI=3 for each virus) was used (Figure 3A and B), and they significantly decreased as the time interval between each infecting virus increased (Figure 4A,B, and C; Figure 5A,B and C). Results from the super infection assays on Vero cells (Additional file 1: Figure S1A and B), known to be interferon defective [18], indicate that the low co-infection rates observed in DF1 cells are due to super infection exclusion between NDV strains LaSota and B1 and not due to an antiviral response of the host cells generated against the primary infection. It is important to note that in addition to specific viral mechanisms that may contribute to super infection exclusion (i.e. neuraminidase activity of the HN protein), the co-infection rates are likely affected by other factors *in vivo,* including strain-specific cell and tissue tropism, the time interval between infection with different strains and the presence of predisposing immunity.

In the future, it will be interesting to use the recombinant virus generated here (rLS-RFP) to determine the mechanism responsible for super infection exclusion in NDV and to assess the potential for recombination among different NDV strains. Complete genome sequencing following super infection experiments will allow us to determine if recombination can indeed occur in NDV and how frequent it may be.

## Conclusions

In summary, the present study describes the characterization of a recombinant NDV strain encoding a red fluorescent protein that was used in co-infection studies. We have shown that NDV strains LaSota and B1 are able to co-infect host cells *in vitro,* however the co-infection rates are low and they decrease as the time between primary infection and super infection increases.

## Abbreviations

ND: Newcastle disease; NDV: Newcastle disease virus; APMV-1: Avian paramyxovirus type 1; [−] ssRNA: Non-segmented single-stranded, negative sense RNA; NP: Nucleoprotein; P: Phosphoprotein; M: Matrix protein; F: Fusion protein; HN: Hemagglutinin-neuraminidase; L: RNA-dependent RNA polymerase; mRNA: Messenger RNA; RFP: Red fluorescent protein; ICPI: Intracerebral pathogenicity index; MDT: Mean death time; CPE: Cytopathic effect; TCID50: Tissue culture infectious dose 50; DMEM: Dulbecco’s modified eagle medium; FBS: Fetal bovine serum; AF: Allantoic fluid; SPF: Specific pathogen free; MVA/T7: Modified vaccinia virus Ankara/T7; GS: Gene start; GE: Gene end; ORF: Open reading frame; HA: Hemagglutination; MOI: Multiplicity of infection.

## Competing interests

The authors declare no competing interest.

## Authors’ contributions

JL constructed the recombinant virus and performed the co-infection studies. HH contributed for recombinant virus construction. QY contributed and coordinated the recombinant virus construction. DGD conceived the co-infection studies. DL participated on experimental design. PJM coordinated the research. All authors have read and approved the final manuscript.

## Supplementary Material

Additional file 1 **Figure S1.** Co-infection of Vero cells with rB1-GFP and rLS-RFP. (A) Vero cells were infected with rLS-RFP (MOI=3) and super infected with rB1-GFP (MOI=3) at 3 h post primary infection. At 24h post infection, cells were examined under a fluorescence microscope. a. red channel; b. green channel; c. merged green, and red channels. (B) Vero cells were infected with rB1-GFP and super infected with rLS-RFP at 12 h post primary infection. At 24h post infection, cells were examined under a fluorescence microscope. a. green channel; b. red channel; c. merged green and red channels. Click here for file
