# Supplementary figures and images for: Generation and characterization of a recombinant Newcastle disease virus expressing the red fluorescent protein for use in co-infection studies
Source: Virol J. 2012 Oct 3;9:227. doi: 10.1186/1743-422X-9-227 (PMC3502164; doi:10.1186/1743-422X-9-227)

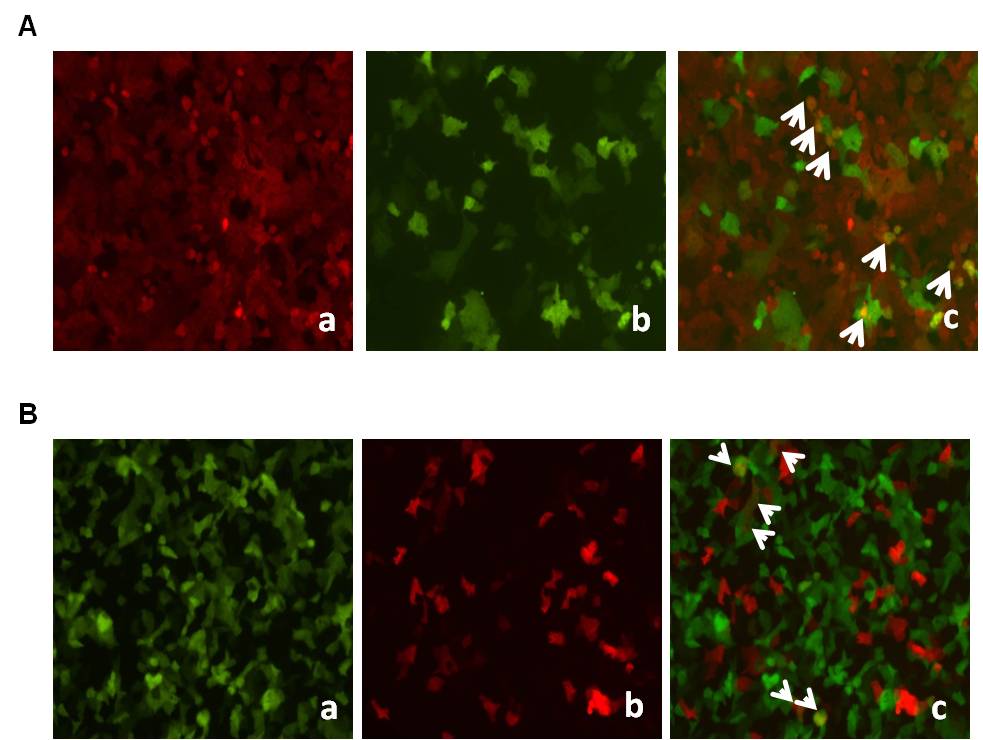

Supplement: Additional file 1 — Figure S1. Co-infection of Vero cells with rB1-GFP and rLS-RFP. (A) Vero cells were infected with rLS-RFP (MOI=3) and super infected with rB1-GFP (MOI=3) at 3 h post primary infection. At 24h post infection, cells were examined under a fluorescence microscope. a. red channel; b. green channel; c. merged green, and red channels. (B) Vero cells were infected with rB1-GFP and super infected with rLS-RFP at 12 h post primary infection. At 24h post infection, cells were examined under a fluorescence microscope. a. green channel; b. red channel; c. merged green and red channels. [file 1743-422X-9-227-S1.jpeg]
